# A phase 3 study (PATHWAY) of palbociclib plus tamoxifen in patients with HR-positive/HER2-negative advanced breast cancer

**DOI:** 10.1038/s41523-024-00684-w

**Published:** 2024-08-22

**Authors:** Emi Noguchi, Takashi Yamanaka, Hirofumi Mukai, Naohito Yamamoto, Chi-Feng Chung, Yen-Shen Lu, Dwan-Ying Chang, Joohyuk Sohn, Gun Min Kim, Kyung-Hun Lee, Soo-Chin Lee, Tsutomu Iwasa, Hiroji Iwata, Kenichi Watanabe, Kyung Hae Jung, Yuko Tanabe, Seok Yun Kang, Hiroyuki Yasojima, Kenjiro Aogi, Eriko Tokunaga, Sung Hoon Sim, Yoon Sim Yap, Koji Matsumoto, Ling-Ming Tseng, Yoshiko Umeyama, Kazuki Sudo, Yuki Kojima, Tomomi Hata, Aya Kuchiba, Taro Shibata, Kenichi Nakamura, Yasuhiro Fujiwara, Kenji Tamura, Kan Yonemori

**Affiliations:** 1 Department of Medical Oncology, National Cancer Center Hospital, Tokyo, Japan; 2 Department of Breast Surgery and Oncology, Kanagawa Cancer Center, Yokohama, Japan; 3 Department of Medical Oncology, National Cancer Center Hospital East, Kashiwa, Japan; 4 Department of Breast Surgery, Chiba Cancer Center Hospital, Chiba, Japan; 5 Koo Foundation Sun Yat-Sen Cancer Center, Taipei, Taiwan; 6 Department of Oncology, National Taiwan University Hospital, Taipei, Taiwan; 7 Division of Medical Oncology, Department of Internal Medicine, Yonsei Cancer Center, Yonsei University College of Medicine, Seoul, Republic of Korea; 8 Department of Internal Medicine, Seoul National University Hospital, Cancer Research Institute, Seoul National University, Seoul, Republic of Korea; 9 Department of Haematology-Oncology, National University Cancer Institute, Singapore, Singapore; 10 Department of Medical Oncology, Faculty of Medicine, Kindai University, Osaka, Japan; 11 Department of Breast Oncology, Aichi Cancer Center Hospital, Nagoya, Japan; 12 Department of Breast Surgery, National Hospital Organization Hokkaido Cancer Center, Sapporo, Japan; 13 Department of Oncology, Asan Medical Center, University of Ulsan College of Medicine, Seoul, Republic of Korea; 14 Department of Medical Oncology, Toranomon Hospital, Tokyo, Japan; 15 Department of Hematology-Oncology, Ajou University School of Medicine, Suwon, Republic of Korea; 16 Departments of Surgery and Breast Oncology, National Hospital Organization Osaka National Hospital, Osaka, Japan; 17 Department of Breast Oncology, National Hospital Organization Shikoku Cancer Center, Matsuyama, Japan; 18 Department of Breast Oncology, National Hospital Organization Kyushu Cancer Center, Fukuoka, Japan; 19 Center for Breast Cancer, National Cancer Center, Goyang-Si, Republic of Korea; 20 Division of Medical Oncology, National Cancer Centre Singapore, Singapore, Singapore; 21 Hyogo Cancer Center Division of Medical Oncology, Akashi, Japan; 22 Division of General Surgery, Department of Surgery, Taipei Veterans General Hospital, Taipei, Taiwan; 23 Pfizer R&D Japan, Tokyo, Japan; 24 International Trial Management Section, Research Management Division, Clinical Research Support Office, National Cancer Center Hospital, Tokyo, Japan; 25 Biostatistics Section, Clinical Research Support Office National Cancer Center Hospital/Biostatistics Division, Center for Research Administration and Support, National Cancer Center, Tokyo, Japan; 26 Department of International Clinical Development, National Cancer Center Hospital, Tokyo, Japan

**Keywords:** Breast cancer, Targeted therapies

## Abstract

Palbociclib combined with endocrine therapy is approved for treating patients with hormone-receptor-positive/human epidermal growth factor receptor 2-negative (HR+/HER2−) advanced breast cancer; however, data on palbociclib combined with tamoxifen are limited. We investigated the efficacy and safety of palbociclib–tamoxifen in patients with HR+/HER2− advanced breast cancer. This double-blind phase 3 study included 184 women who were randomly assigned 1:1 to receive palbociclib–tamoxifen or placebo–tamoxifen. Pre/perimenopausal women also received goserelin. The primary endpoint was investigator-assessed progression-free survival (PFS). Secondary endpoints included overall survival (OS) and safety. Median PFS was 24.4 months (95% confidence interval [CI], 13.1–32.4) with palbociclib–tamoxifen and 11.1 months (95% CI, 7.4–14.6) with placebo–tamoxifen (hazard ratio [HR], 0.60; 95% CI, 0.43–0.85; *P* = 0.002). Palbociclib–tamoxifen improved PFS in patients who were treated with first-line or second-line endocrine therapy and pre-, peri-, and postmenopausal patients. Though OS data are still immature (median not reached in both groups), an overall risk reduction of 27% (HR, 0.73; 95% CI, 0.44–1.21) with palbociclib–tamoxifen was observed at the time of PFS analysis. The most common grade 3/4 adverse event with palbociclib–tamoxifen was neutropenia (89.0% [none were febrile] versus 1.1% with placebo–tamoxifen). There were no deaths owing to adverse events in either group. Among patients with HR+/HER2− advanced breast cancer, palbociclib–tamoxifen resulted in significantly longer PFS than tamoxifen alone. Early OS data showed a trend favoring palbociclib–tamoxifen. Trial registration: ClinicalTrials.gov number, NCT03423199. Study registration date: February 06, 2018.

## Introduction

The tumor characteristics of breast cancer in premenopausal women differ from those of postmenopausal women. Premenopausal patients tend to be diagnosed with more advanced cancer that is associated with worse clinical outcomes^1^. In Asian countries, the incidence of breast cancer is increasing, with a higher proportion of pre/perimenopausal cases than in Western countries^2,3^. However, treatment options for pre/perimenopausal women with breast cancer remain limited. When this study began in 2018, the combination of tamoxifen and luteinizing hormone-releasing hormone agonists (such as goserelin) for ovarian function suppression was one of the treatment options for first-line endocrine therapy (ET) for pre/perimenopausal patients with hormone receptor-positive (HR+)/human epidermal growth factor receptor 2–negative (HER2−) advanced breast cancer^4^. Tamoxifen was also a treatment option for postmenopausal patients with advanced breast cancer after treatment with aromatase inhibitors (AIs) or when AIs were intolerable^5^. Though ET remains the mainstay of treatment, there is inevitable resistance after a period of time, which has led to the development of targeted therapies^6^.

The cyclin-dependent kinase 4/6 (CDK4/6)-cyclin D axis is hyperactive in HR+/HER2− breast cancer^7^. Two pivotal studies, PALOMA-2 and PALOMA-3, demonstrated that adding the CDK4/6 inhibitor palbociclib to ET resulted in prolonged progression-free survival (PFS) over ET alone in patients with HR+/HER2− breast cancer^8,9^. CDK4/6 inhibitors, such as palbociclib, in combination with ET have become an established therapeutic approach for HR+/HER2− breast cancer^7^. Despite significant progress with CDK4/6 inhibitors, no phase 3 studies have evaluated the efficacy and safety of palbociclib in combination with tamoxifen in patients with HR+/HER2− advanced breast cancer regardless of menopausal status.

Here we report results from the PATHWAY trial (NCCH1607), which has investigated the benefit of adding palbociclib to tamoxifen in patients with HR+/HER2− advanced breast cancer, in both pre-, peri-, and postmenopausal patients versus tamoxifen alone.

## Results

### Patient characteristics

**Fig. 1:**
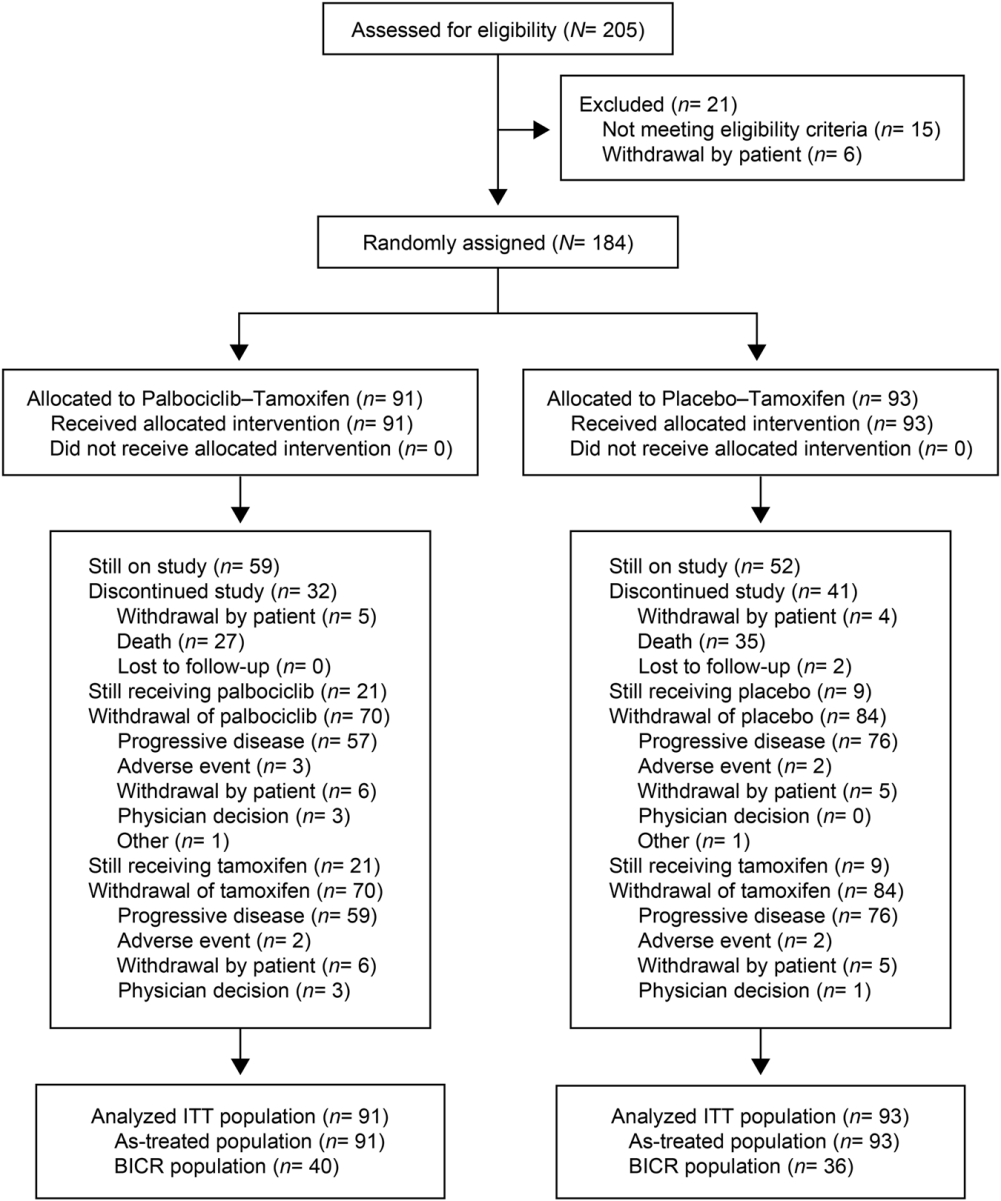
CONSORT diagram. ITT intent-to-treat; BICR blinded independent central review.

From February 15, 2018, to July 30, 2019, 184 patients were randomly assigned to receive palbociclib–tamoxifen (91 patients) or placebo–tamoxifen (93 patients) at 22 sites in 4 countries. All randomly assigned patients were treated (Fig. 1). The baseline demographic and clinical characteristics were balanced between the 2 treatment groups (Table 1). Overall, approximately 72% of the patients were postmenopausal and 61% received study treatment as first-line therapy.

**Table 1:** Demographic and baseline clinical characteristics

Characteristic	Palbociclib–Tamoxifen Group (*n* = 91)	Placebo–Tamoxifen Group (*n* = 93)
Age, median (range), years	60 (33–82)	61 (35–83)
Weight, median (range), kg	53.9 (32.5–89.7)	56.0 (31.7–96.0)
BMI, median (range), kg/m^2^	21.9 (14.3–38.8)	23.2 (13.1–35.3)
Geographical region, n (%)		
Japan	69 (75.8)	49 (52.7)
Republic of Korea	12 (13.2)	19 (20.4)
Taiwan	6 (6.6)	18 (19.4)
Singapore	4 (4.4)	7 (7.5)
ECOG performance status, n (%)		
0	72 (79.1)	61 (65.6)
1	19 (20.9)	32 (34.4)
Endocrine therapy^a^, n (%)		
First-line endocrine therapy	56 (61.5)	56 (60.2)
Second-line endocrine therapy	35 (38.5)	37 (39.8)
Menopausal status^**a**^, n (%)		
Pre/perimenopausal	25 (27.5)	27 (29.0)
Postmenopausal	66 (72.5)	66 (71.0)
Visceral metastases, n (%)		
Yes	40 (44.0)	50 (53.8)
No	51 (56.0)	43 (46.2)
Bone-only metastasis, n (%)		
Yes	9 (9.9)	7 (7.5)
No	82 (90.1)	86 (92.5)
Prior cancer-related radiotherapy, n (%)		
Yes	34 (37.4)	44 (47.3)
No	57 (62.6)	49 (52.7)
Prior primary diagnosis cancer-related surgery, n (%)		
Yes	48 (52.7)	53 (57.0)
No	43 (47.3)	40 (43.0)
Recurrence type, n (%)		
Locoregional	4 (4.4)	3 (3.2)
Local	3 (3.3)	3 (3.2)
Regional	1 (1.1)	5 (5.4)
Distant	51 (56.0)	55 (59.1)
Newly diagnosed	32 (35.2)	27 (29.0)
Biomarker status, n (%)^b^		
* PIK3CA* mutation		
Positive	28 (30.8)	24 (25.8)
Negative	62 (68.1)	66 (71.0)
* ESR1* mutation		
Positive	12 (13.2)	9 (9.7)
Negative	78 (85.7)	81 (87.1)
* BRCA1*/2 mutation		
Positive	5 (5.5)	3 (3.2)
Negative	85 (93.4)	87 (93.5)

*BMI* body mass index, *BRCA1/2* breast cancer 1 or 2 gene, *ECOG* Eastern Cooperative Oncology Group, *ESR1* estrogen receptor 1 gene, *PIK3CA* phosphatidylinositol 4,5-bisphosphate 3-kinase catalytic subunit alpha gene.

^a^Based on the registration system.

^b^Biomarkers were assessed in 90 patients each in Palbociclib-Tamoxifen and Placebo-Tamoxifen groups.

### Progression-free survival

**Fig. 2:**
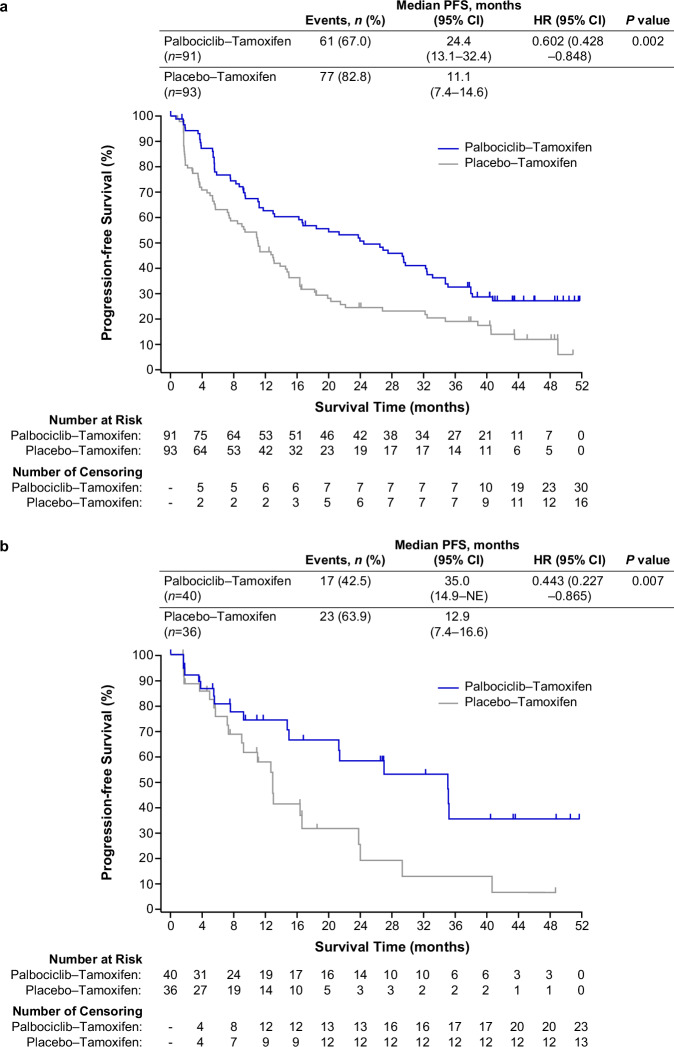
Progression-free survival. **a** Progression-free survival by investigator assessment. **b** Progression-free survival by blinded independent central review. The tick marks indicate censored data. CI confidence interval; HR hazard ratio; NE not estimable; PFS progression-free survival.

At the data cut-off date (September 15, 2022), 138 PFS events had occurred after a median duration of follow-up of 40.9 months for censored patients. The median PFS was 24.4 months (95% confidence interval [CI], 13.1–32.4) with palbociclib–tamoxifen, compared with 11.1 months (95% CI, 7.4–14.6) with placebo–tamoxifen (hazard ratio [HR], 0.60 [95% CI 0.43–0.85]; *P* = 0.002; Fig. 2a). The median PFS as assessed by blinded independent central review (BICR) was 35.0 months (95% CI, 14.9–not estimable [NE]) with palbociclib–tamoxifen, compared with 12.9 months (95% CI, 7.4–16.6) with placebo–tamoxifen (HR, 0.44 [95% CI 0.23–0.87]; *P* = 0.007; Fig. 2b).

**Fig. 3:**
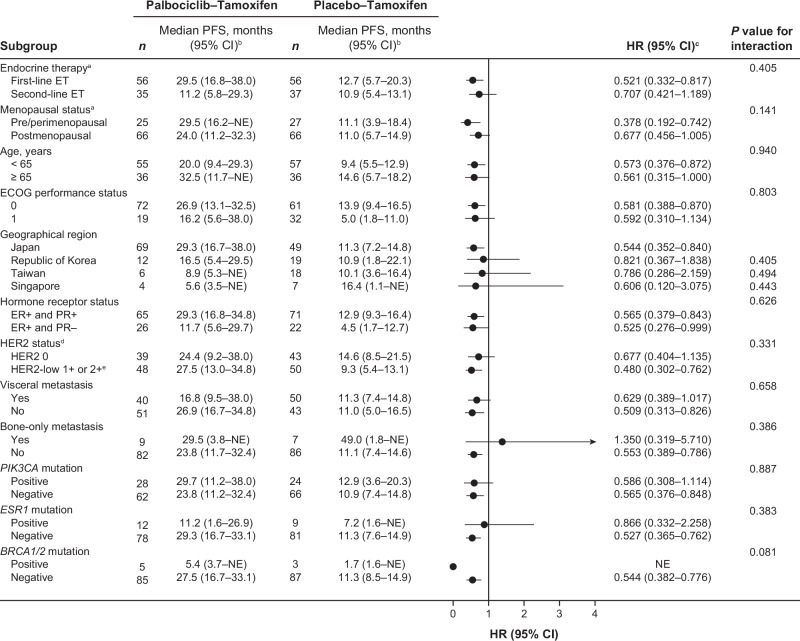
Progression-free survival based on investigator assessment for all subgroups. ^a^Based on the registration system. ^b^Brookmeyer and Crowley method. ^c^HR and the corresponding 2-sided 95% CI for the palbociclib group relative to the placebo group were calculated by unstratified Cox proportional hazards model. ^d^Categorized by IHC. Tests by IHC were not conducted in 4 patients in the Palbociclib-Tamoxifen group. ^e^All patients with HER2 2+ were negative by in situ hybridization. *BRCA1/2* breast cancer 1 or 2 gene; CI confidence interval; ECOG Eastern Cooperative Oncology Group; ER estrogen receptor; *ESR1* estrogen receptor 1 gene; ET endocrine therapy; HER2 human epidermal growth factor receptor 2-negative; HR hazard ratio; IHC immunohistochemistry; NE not estimable; PFS progression-free survival; *PIK3CA* phosphatidylinositol 4,5-bisphosphate 3-kinase catalytic subunit alpha gene; PR progesterone receptor.

**Fig. 4:**
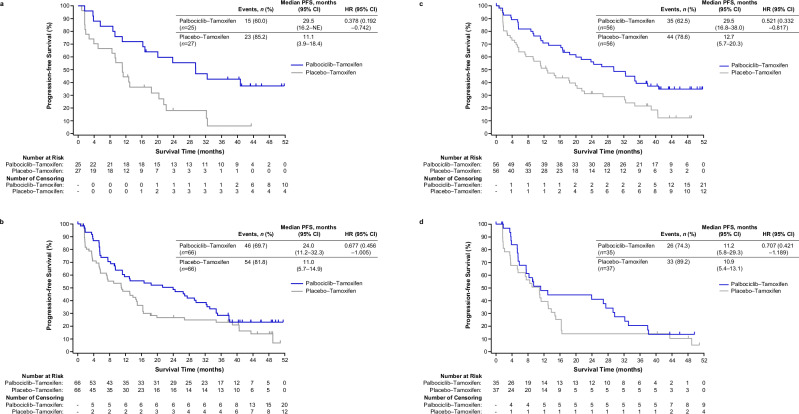
Progression-free survival based on investigator assessment (subgroup analysis). **a** Progression-free survival in pre/perimenopausal patients**. b** Progression-free survival in postmenopausal patients. **c** Progression-free survival in patients treated with first-line endocrine therapy. **d** Progression-free survival in patients treated with second-line endocrine therapy. The tick marks indicate censored data. CI confidence interval; HR hazard ratio; NE not estimable; PFS progression-free survival.

In subgroup analyses, PFS favored palbociclib–tamoxifen over placebo–tamoxifen treatment across all subgroups, except for patients with bone-only metastasis. No treatment interactions were observed in all subgroups (Fig. 3). The small number of patients with bone-only metastasis (palbociclib–tamoxifen: 9 and placebo–tamoxifen: 7) made results difficult to interpret. In the pre/perimenopausal subgroup, median PFS was longer with palbociclib–tamoxifen (29.5 months; 95% CI, 16.2–NE) compared with placebo–tamoxifen (11.1 months; 95% CI, 3.9–18.4; HR, 0.38; 95% CI, 0.19–0.74) (Fig. 4a). Similarly, in the postmenopausal subgroup, median PFS was longer with palbociclib–tamoxifen (24.0 months; 95% CI, 11.2–32.3) than with placebo–tamoxifen (11.0 months; 95% CI, 5.7–14.9; HR, 0.68; 95% CI, 0.46–1.01) (Fig. 4b).

The first-line ET subgroup had a HR of 0.52 (95% CI, 0.33–0.82) with a median PFS of 29.5 months (95% CI, 16.8–38.0) with palbociclib–tamoxifen and 12.7 months (95% CI, 5.7–20.3) with placebo–tamoxifen (Fig. 4c). The second-line ET subgroup had a HR of 0.71 (95% CI, 0.42–1.19), median PFS was 11.2 months (95% CI, 5.8–29.3) with palbociclib–tamoxifen and 10.9 months (95% CI, 5.4–13.1) with placebo–tamoxifen (Fig. 4d).

### Overall survival

**Fig. 5:**
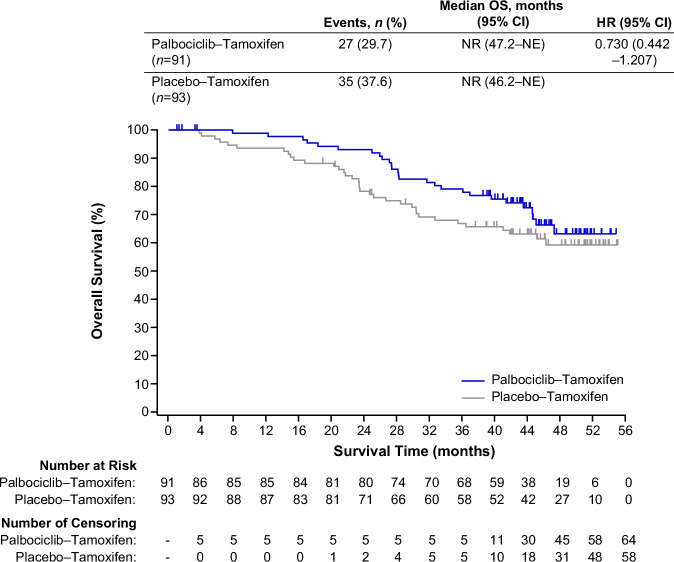
Kaplan–Meier plot of overall survival. Tick marks represent data censored at the last time the patient was known to be alive. CI confidence interval; HR hazard ratio; NE not estimable; NR not reached.

The median overall survival (OS) was not reached in either the palbociclib–tamoxifen (95% CI, 47.2–NE) or the placebo–tamoxifen group (95% CI, 46.2–NE), with a HR of 0.73 (95% CI, 0.44–1.21; Fig. 5). The estimated survival rates (95% CI) in the palbociclib–tamoxifen and placebo–tamoxifen groups were 98.8% (92.0–99.8) and 93.5% (86.2–97.0) at 1 year, 93.0% (85.1–96.8) and 78.3% (68.4–85.4) at 2 years, and 79.1% (68.9–86.3) and 66.8% (56.1–75.5) at 3 years. Double-blinding of the study will be maintained until the final OS analysis.

### Objective response

The objective response rate in the intent-to-treat population was higher in the palbociclib–tamoxifen group at 44.0% (95% CI, 33.6–54.8) compared with 28.0% (95% CI, 19.1–38.2) in the placebo–tamoxifen group (Supplementary Table 1). Consistent with these findings, patients who had measurable disease had a more favorable response rate with palbociclib–tamoxifen at 53.4% (95% CI, 41.4–65.2) than with placebo–tamoxifen (34.2%; 95% CI, 23.7–46.0).

### Clinical benefit

The clinical benefit rate was greater with palbociclib–tamoxifen treatment (75.8%; 95% CI, 65.7–84.2) versus placebo–tamoxifen (61.3%; 95% CI, 50.6–71.2).

### Exposure and safety

The median (range) duration of treatment with palbociclib was 19.9 months (0.2–52.2), whereas placebo was administered for a median of 10.8 months (0.7–50.9). The median (range) duration of treatment with tamoxifen was 20.2 months (0.2–52.4) in the palbociclib–tamoxifen group and 11.0 months (1.0–50.9) in the placebo–tamoxifen group. There were 61 (67.0%) patients who needed at least 1 dose reduction of palbociclib. Of those patients, 38 (41.8%) had 2 dose reductions from 125 mg to 75 mg. The median relative dose intensity of palbociclib was 68.8% in the palbociclib–tamoxifen group and 98.7% for placebo in the placebo–tamoxifen group. The median relative dose intensities of tamoxifen were 99.2% and 99.8% in the palbociclib–tamoxifen and placebo–tamoxifen groups, respectively.

Adverse events (AEs) were reported in 89 patients in the palbociclib–tamoxifen group (97.8%) and 81 patients in the placebo–tamoxifen group (87.1%; Table 2), grade 3/4 AEs were reported in 85 (93.4%) and 19 (20.4%) patients. The most common grade 3/4 AEs were neutropenia (89.0%) and leukopenia (28.6%; none were grade 4 AEs) in the palbociclib–tamoxifen group. Febrile neutropenia was not reported in either treatment group. Grade 5 AEs (deaths) were not reported in either treatment group.

**Table 2:** Adverse events reported in ≥ 10% of patients in either group, by severity grade and type

Adverse event	Palbociclib–Tamoxifen Group (*n* = 91)			Placebo–Tamoxifen Group (*n* = 93)		
	Any Grade, *n* (%)	Grade 3, *n* (%)	Grade 4, *n* (%)	Any Grade, *n* (%)	Grade 3, *n* (%)	Grade 4, *n* (%)
Any adverse events	89 (97.8)	69 (75.8)	16 (17.6)	81 (87.1)	17 (18.3)	2 (2.2)
Neutropenia^a^	83 (91.2)	69 (75.8)	12 (13.2)	2 (2.2)	1 (1.1)	0
Infections^b^	45 (49.5)	5 (5.5)	1 (1.1)	31 (33.3)	4 (4.3)	0
Leukopenia^c^	44 (48.4)	26 (28.6)	0	2 (2.2)	0	0
Stomatitis^d^	34 (37.4)	0	0	11 (11.8)	0	0
Thrombocytopenia^e^	31 (34.1)	3 (3.3)	2 (2.2)	2 (2.2)	0	0
Anemia^f^	28 (30.8)	6 (6.6)	0	9 (9.7)	1 (1.1)	0
Rash^g^	28 (30.8)	0	0	7 (7.5)	0	0
Constipation	20 (22.0)	0	0	8 (8.6)	1 (1.1)	0
Aspartate aminotransferase increased	18 (19.8)	3 (3.3)	0	5 (5.4)	1 (1.1)	0
Pyrexia	18 (19.8)	0	0	6 (6.5)	0	0
Alanine aminotransferase increased	17 (18.7)	4 (4.4)	1 (1.1)	6 (6.5)	1 (1.1)	0
Back pain	14 (15.4)	1 (1.1)	0	9 (9.7)	1 (1.1)	0
Arthralgia	13 (14.3)	1 (1.1)	0	15 (16.1)	0	0
Pruritus	13 (14.3)	0	0	1 (1.1)	0	0
Fatigue	11 (12.1)	0	0	9 (9.7)	1 (1.1)	0
Nausea	11 (12.1)	0	0	11 (11.8)	0	0
Headache	10 (11.0)	0	0	7 (7.5)	0	0
Vomiting	10 (11.0)	1 (1.1)	0	3 (3.2)	1 (1.1)	0
Cough	8 (8.8)	0	0	15 (16.1)	0	0
Hot flash	7 (7.7)	0	0	13 (14.0)	0	0

Adverse events were graded by CTCAE v4.0.

Patient with >1 adverse event within the same level of MedDRA term is counted as 1 at its maximum grade.

*CTCAE* common terminology criteria for adverse events, *MedDRA* medical dictionary for regulatory activities, *PT* preferred term.

^a^Neutropenia included events with the PTs of neutrophil count decreased and neutropenia.

^b^Infections included events with the PTs of nasopharyngitis, upper respiratory tract infection, cellulitis, cystitis, COVID-19, hordeolum, influenza, oral herpes, pneumonia, herpes zoster, tinea infection, COVID-19 pneumonia, conjunctivitis, dermatophytosis of nail, erysipelas, fungal skin infection, herpes simplex, impetigo, omphalitis, otitis externa, paronychia, parotitis, periodontitis, pharyngitis, pneumonia bacterial, septic shock, sinusitis, suspected COVID-19, urinary tract infection, brain abscess, bronchitis, denture stomatitis, gastroenteritis, helicobacter gastritis, herpes virus infection, myringitis, osteomyelitis, periorbital infection, pulpitis dental, sialadenitis, soft tissue infection, tinea pedis, tooth abscess, and vaginal infection.

^c^Leukopenia included events with the PT of white blood cell count decreased.

^d^Stomatitis included events with the PTs of stomatitis, mucosal inflammation, oropharyngeal pain, cheilitis, glossitis, mouth ulceration, and glossodynia.

^e^Thrombocytopenia included events with the PT of platelet count decreased.

^f^Anemia included events with the PTs of anemia and hemoglobin decreased.

^g^Rash included events with the PTs of rash, rash maculo-papular, and rash erythematous.

Serious AEs were reported in 17.6% of patients treated with palbociclib–tamoxifen and in 15.1% of patients treated with placebo–tamoxifen; 2.2% and 3.2% were reported as treatment-related serious AEs (Supplementary Table 2). AEs leading to treatment discontinuation were reported in 3.3% and 2.2% of patients in the palbociclib–tamoxifen and placebo–tamoxifen groups, respectively (Supplementary Table 3). One patient in each treatment group experienced a greater than 60 msec QTcF prolongation from baseline; there were no patients with postbaseline QTcF of more than 480 msec in either treatment group.

### Subsequent line of therapy

At the data cut-off date, 70 and 84 patients had discontinued treatment with palbociclib–tamoxifen and placebo–tamoxifen, respectively. Subsequent anticancer therapy was reported in 64 (70.3%) and 80 (86.0%) patients in the palbociclib–tamoxifen and placebo–tamoxifen groups, respectively (Supplementary Table 4). CDK4/6 inhibitors as first subsequent therapy were received by 11 (12.1%) and 34 patients (36.6%), respectively.

### Biomarkers

PFS was compared by baseline mutational status for specific genes in both treatment groups (Fig. 3). Regardless of *PIK3CA* mutation status, palbociclib–tamoxifen treatment trended in favor of improved PFS over placebo–tamoxifen. The small number of patients with *ESR1* (palbociclib–tamoxifen: 12, placebo–tamoxifen: 9) or *BRCA1/2* mutations (palbociclib–tamoxifen: 5, placebo–tamoxifen: 3) precluded comparison of PFS between the 2 treatment groups.

## Discussion

PATHWAY is a phase 3 trial evaluating efficacy and safety of palbociclib in combination with tamoxifen. This trial achieved its primary endpoint, demonstrating both statistically significant and clinically meaningful improvements in PFS for patients with HR+/HER2− advanced breast cancer treated with palbociclib–tamoxifen compared with placebo–tamoxifen. A clinical benefit in PFS with palbociclib–tamoxifen treatment was observed both in patients who were treated with first- and second-line ET and regardless of menopausal status.

This study included a heterogeneous population of pre- and postmenopausal patients receiving first-line ET as well as those resistant to AIs and receiving second-line ET. Although the number of pre/perimenopausal patients in this study is small, there was a 62% lower relative risk of progression or death with palbociclib–tamoxifen in this population compared with those receiving placebo–tamoxifen (HR, 0.38 [95% CI, 0.19–0.74]). In MONALEESA-7, a phase 3, placebo-controlled trial which included 672 premenopausal women with advanced, HR-positive breast cancer, the HR of PFS in the subgroup of ribociclib in combination with tamoxifen was 0.59 (95% CI, 0.39–0.88)^10^. However, cross-trial comparisons should be interpreted with caution because of differences in study designs and patient populations. Ribociclib is approved in the Republic of Korea, Taiwan, and Singapore, but its development has been halted in Japan due to dose-limiting toxicities that led to a different recommended phase 2 dose^11^. Since tamoxifen remains a valid treatment option for both pre-, peri-, and postmenopausal women with HR+/HER2− advanced breast cancer, given the higher risk of prolonged QTcF with tamoxifen–ribociclib and higher risk of venous thromboembolic events with tamoxifen–abemaciclib^10,12^, new treatment options that reduce the risk of serious AEs would be of clinical importance. Beyond clinical trials, the incidence of thromboembolic events with CDK4/6 inhibitors remains a topic of research interest. In a real-world study of 266 patients with breast cancer receiving CDK4/6 inhibitors, thromboembolic events including arterial and venous events were more frequent with palbociclib and ribociclib than with abemaciclib; however, palbociclib comprised the vast majority of CDK 4/6 inhibitors in the study, making comparisons between the agents challenging^13^. Furthermore, the incidence of venous thromboembolic events reported in this study was higher than that observed in a meta-analysis of randomized controlled trials in patients treated with CDK4/6 inhibitors^14^. Therefore, this finding needs to be validated by other real-world data; nevertheless, physicians monitor thromboembolism in every patient who receives a CDK4/6 inhibitor.

Though, in postmenopausal patients with advanced breast cancer, the use of tamoxifen as the first-line ET backbone is not common and limited, the results of the SONIA trial suggest that an AI alone may remain a treatment option for first-line treatment^15^. Therefore, for those who have failed AI, or who are unable to maintain adherence due to side effects such as arthralgia and osteoporosis, this study showed the efficacy of palbociclib plus tamoxifen in postmenopausal breast cancer and supports the use of this combination therapy as one of the treatment options.

The PATHWAY study had limited enrollment of premenopausal patients who have received AI plus ovarian function suppression, either as adjuvant ET or as first-line ET in advanced setting. However, following the results of SOFT and TEXT trials^16,17^, the use of AI plus ovarian function suppression as adjuvant ET in premenopausal patients has been increasing, particularly for those with high risk of recurrence. Since incomplete ovarian function suppression remains a concern for some patients^18^, tamoxifen plus ovarian function suppression plus palbociclib is a reasonable choice once patients develop disease recurrence with AI plus ovarian function suppression. In addition, given that palbociclib and tamoxifen appear to be a safe combination therapy, it could be used as a bridge when starting ovarian suppression and waiting for the ovaries to be suppressed (prior to being able to initiate treatment with an AI).

Although fulvestrant was a strongly recommended treatment for patients previously treated with ET, tamoxifen, too, remained a treatment option based on patient preference. Hence, patients who were being considered for tamoxifen were included in this study. In addition, several agents such as alpelisib or capivasertib are now being available for use in combination with fulvestrant in the subsequent line after treatment with ET or ET plus CDK4/6 inhibitor^19–21^. The combination of palbociclib and tamoxifen provides value in expanding the treatment options for HR+/HER2− advanced breast cancer beyond palbociclib combinations with AI or fulvestrant.

The OS data had not matured, and median OS was not reached in either treatment group at the time of the PFS analysis; however, a trend favoring the palbociclib–tamoxifen group was observed. The clinical benefit of palbociclib in combination with tamoxifen was also maintained across all secondary endpoints evaluated including objective response and clinical benefit response, confirming the robustness of the results.

Attributable toxicities with palbociclib in combination with tamoxifen were manageable with dosing interruptions and/or dose reduction of palbociclib. The AEs reported were generally consistent with the known safety profile of palbociclib in combination with other ET. There were no unexpected major safety findings in this study population. Neutropenia was the most common AE among patients receiving palbociclib in this study. The incidence of neutropenia in this study were similar to those reported in the Asian-race subgroup analysis from PALOMA-2 and PALOMA-3^22,23^. QTcF interval prolongation with palbociclib–tamoxifen was not frequent.

In PALOMA-3, a clinical benefit was reported with palbociclib–fulvestrant regardless of *PIK3CA-* and *ESR1*-mutation status^24,25^. Although a clinical benefit was observed with palbociclib–tamoxifen treatment regardless of *PIK3CA* mutation status, interpretation by *ESR1-* or *BRCA1*/2*-*mutation status is limited by the small sample size of patients.

A possible limitation is that this study included only patients of Asian origin; therefore, caution is needed when considering how these results may apply to other racial groups. However, no significant racial and ethnic differences in efficacy parameters have been reported in previous international studies of palbociclib^8,9,22,23^.

In conclusion, data from the PATHWAY trial showed the clinical benefit of treatment with palbociclib in combination with tamoxifen in patients with HR+/HER2− advanced breast cancer. Although OS results are not yet mature, early OS data showed a trend favoring palbociclib in combination with tamoxifen over placebo with tamoxifen.

## Methods

### Trial design

PATHWAY (ClinicalTrials.gov number: NCT03423199; study registration date: February 06, 2018) is an international (Japan, Republic of Korea, Taiwan, and Singapore), multicenter, randomized, double-blind, placebo-controlled, phase 3 clinical trial. The protocol was approved by the institutional review board at each trial site, and all patients provided written informed consent.

### Patients

Pre-, peri-, or postmenopausal women with locally advanced or metastatic HR+/HER2− breast cancer were eligible if they were candidates to receive tamoxifen as first-line or second-line ET for advanced disease. Patients were excluded if they had received prior treatment with a CDK4/6 inhibitor or tamoxifen. Patients who had disease progression more than 12 months after the completion of adjuvant therapy with tamoxifen were eligible. One previous line of chemotherapy for advanced disease was allowed.

### Randomization and treatments

Patients were randomly assigned 1:1 to receive either palbociclib–tamoxifen or placebo–tamoxifen ± goserelin (Supplementary Fig. 1). Patients were stratified by treatment history with ET (first-line or second-line) and by menopausal status (pre/perimenopausal versus postmenopausal) at randomization. Study treatment that was given after recurrence during treatment or within 12 months after completion of adjuvant ET was defined as second-line ET. Patients received either palbociclib (starting dose, 125 mg/day) or placebo orally once daily on day 1 to day 21 followed by 7 days off-treatment for each 28-day cycle, plus tamoxifen 20 mg orally once daily (continuously). Pre/perimenopausal women additionally received goserelin subcutaneously 3.6 mg given every 4 weeks, or a long-acting form 10.8 mg given every 12 weeks.

Dose adjustment was permitted for palbociclib/placebo only. Dose reduction of palbociclib by 1 dose level (to 100 mg/day), and, if needed, by 2 dose levels (to 75 mg/day) was recommended depending on type and severity of the toxicity. Patients were to receive assigned treatment until either disease progression, unacceptable toxicity, death, or withdrawal of consent.

### Assessments

Tumor assessments were performed per Response Evaluation Criteria in Solid Tumors version 1.1 at baseline, every 8 weeks for the first 1.5 years, and then every 12 weeks thereafter. Laboratory tests and vital signs were performed on day 1 and day 15 of the first 3 cycles and day 1 of subsequent cycles. AEs were graded with the Common Terminology Criteria for Adverse Events version 4.0. Plasma samples for circulating tumor DNA (ctDNA) analysis were collected on cycle 1 day 1, cycle 2 day 15, and the end of treatment. Mutations in the *PIK3CA*, *ESR1*, and *BRCA1*/2 (somatic or germline mutation) genes were detected using Guardant360 (Guardant Health, Inc., CA, USA).

### Outcome measures

The primary endpoint was investigator-assessed PFS, defined as the time from randomization to radiological or clinical disease progression or death due to any cause, whichever occurred first. Secondary endpoints included OS, objective response, duration of response, clinical benefit (defined as a complete response, partial response, or stable disease for 24 weeks or longer), pharmacokinetics, safety, and patient-reported outcomes. Exploratory endpoint included biomarkers obtained through blood sampling. PFS was also assessed by BICR for a randomly selected subgroup of patients (~40%).

### Trial oversight

The trial was conducted in accordance with the International Council for Harmonisation Good Clinical Practice guidelines and the Declaration of Helsinki. The trial sponsor (National Cancer Center Hospital, Japan; IRB approval number: T4467) and the principal academic investigators designed the trial; Pfizer provided the trial drugs and placebo. Operation of this trial was reported elsewhere^26^. The trial was supervised by institutional review boards in Japan: National Cancer Center Hospital, Aichi Cancer Center Hospital, National Hospital Organization Osaka National Hospital, National Hospital Organization Hokkaido Cancer Center, National Cancer Center Hospital East, National Hospital Organization Shikoku Cancer Center, Chiba Cancer Center, Kanagawa Cancer Center, Toranomon Hospital, Hyogo Cancer Center, Kindai University Hospital, and Kyusyu Cancer Center; Republic of Korea: Severance Hospital, Seoul National University Hospital, Asan Medical Center, National Cancer Center, Seoul National University Bundang Hospital, and Ajou University Hospital; Taiwan: National Taiwan University Hospital, Koo Foundation Sun Yat-Sen Cancer Center, and Taipei Veterans General Hospital; and Singapore: Singhealth Centralised. Data were collected by the sponsor and analyzed in collaboration with the authors. An independent safety monitoring committee reviewed safety data on an ongoing basis. The authors vouch for the accuracy and completeness of the data and for the fidelity of the trial to the protocol.

### Statistical analysis

A total of 138 PFS events were required based on the 1:1 randomization to have an 80% power to detect a 38% reduction in the risk of disease progression or death for the palbociclib–tamoxifen group, with a 1-sided log-rank test at a significance level of 0.025. Assuming a 10% dropout rate, approximately 180 patients were planned to be randomly assigned to a treatment. The intent-to-treat population was the primary population for evaluating all efficacy endpoints and patient characteristics; the as-treated population was the primary population evaluating treatment administration, compliance, and safety.

A stratified log-rank test was used to compare PFS between the 2 treatment groups. The 95% CI of median PFS was calculated by the Brookmeyer and Crowley method. The stratified Cox Proportional hazards model was used to estimate the treatment HR and the corresponding 95% CI. The stratified analysis was performed with three strata: pre/perimenopausal, postmenopausal, and first-line ET, and postmenopausal and second-line ET. The stratified analysis was originally planned with four strata; however, because of the small sample size of three patients in the pre/perimenopausal and second-line ET strata, the statistical analysis plan was updated before the database lock to combine the pre/perimenopausal and first-line ET and pre/perimenopausal and second-line ET strata. Treatment—Factor interactions were explored for the factors used for subgroup analysis in the PFS. The *P* values for interactions were calculated by Cox proportional hazard models including the treatment, factor, and their interaction term.

The current OS outcome was evaluated at the time of PFS analysis. The final OS analysis will be performed at least 3 years from randomization of the last patient. OS was evaluated using a stratified log-rank test with HRs and 95% CIs calculated as described for PFS. Reported *P* values for PFS analyses are 1-sided and *P* values for interactions are 2-sided. All analyses were performed with SAS, version 9.4 or higher (SAS Institute, Cary, NC).

### Supplementary information

Supplementary Appendix

## Data Availability

The datasets used and/or analyzed during the current study are available from the corresponding author on reasonable request and with the permission of National Cancer Center Hospital.
