# Supplementary material for: A phase 3 study (PATHWAY) of palbociclib plus tamoxifen in patients with HR-positive/HER2-negative advanced breast cancer
Source: NPJ Breast Cancer. 2024 Aug 22;10:76. doi: 10.1038/s41523-024-00684-w (PMC11341958; doi:10.1038/s41523-024-00684-w)
Supplement: Supplementary file 1 — Supplementary Appendix [file 41523_2024_684_MOESM1_ESM.pdf]

Supplementary Appendix

Table of contents

List of Collaborators
**Supplementary Figure 1.** Trial design.
**Supplementary Table 1.** Best overall response based on investigator assessment.
**Supplementary Table 2.** Serious adverse events related to study treatment, by severity grade and type.
**Supplementary Table 3.** Adverse events leading to treatment discontinuation, by severity grade and type.
**Supplementary Table 4.** Follow-up anticancer therapy received by 3% or more of patients in either treatment group.
CONSORT Checklist

List of Collaborators

Japan

Principal Investigator<br>(P)：Previous Principal Investigator	Sub-Investigator<br>(P)：Previous Sub-Investigator	Study Conducted at Address(es)
Kan Yonemori<br>Kenji Tamura (P)	Yasuhiro Fujiwara (P)<br>Chikako Shimizu (P)<br>Kan Yonemori (P)<br>Akihiko Shimomura (P)<br>Emi Noguchi (P)<br>Kazuki Sudo<br>Tadaaki Nishikawa<br>Hitomi Okuma<br>Hiromitsu Kitayama (P)<br>Takuji Seo (P)<br>Maki Tanioka (P)<br>Yohei Otake (P)<br>Yuki Kojima (P)<br>Tatsunori Shimoi<br>Toshihiro Okuya (P)<br>Shu Yazaki (P)<br>Yohei Chiba (P)<br>Kasumi Yamamoto (P)<br>Shosuke Kita<br>Chiharu Mizoguchi (P)<br>Momoko Tokura (P)<br>Motoko Arakaki (P)<br>Ayumi Saito<br>Aiko Maejima<br>Asuka Kawachi<br>Rui Kitadai<br>Mai Onishi	National Cancer Center Hospital<br>5-1-1 Tsukiji, Chuo-ku, Tokyo, 104-0045, Japan
Hiroji Iwata	Masataka Sawaki<br>Haruru Kotani<br>Yayoi Adachi (P)<br>Sakura Onishi (P)<br>Madoka Iwase (P)<br>Kayoko Sugino (P)<br>Nanae Horisawa (P)<br>Makiko Mori (P)<br>Mitsuo Terada (P)<br>Masashi Ando<br>Yoshitaka Inaba<br>Yoshiko Murakami (P)<br>Akiyo Yoshimura<br>Naomi Gondo (P)<br>Yuri Ozaki<br>Ayumi Kataoka<br>Yuka Endo<br>Kazuki Nozawa<br>Shoko Sakamoto (P)<br>Masaya Hattori<br>Hosoda Waki	Aichi Cancer Center<br>1-1 Kanokoden, Chikusa-ku, Nagoya, Aichi, 464-8681, Japan

Principal Investigator<br>(P)：Previous Principal Investigator	Sub-Investigator<br>(P)：Previous Sub-Investigator	Study Conducted at Address(es)
Hiroyuki Yasojima<br>Norikazu Masuda (P)	Makiko Mizutani (P)<br>Hiroyuki Yasojima (P)<br>Yoko Otani (P)<br>Nozomi Morikawa (P)<br>Misato Hagi (P)<br>Masayuki Mano<br>Kiyoshi Mori<br>Kumiko Okada<br>Kaori Akazawa	National Hospital Organization<br>Osaka National Hospital<br>2-1-14 Hoenzaka, Chuo-ku,<br>Osaka, 540-0006, Japan
Kenichi Watanabe<br>Masato Takahashi (P)	Kenichi Watanabe (P)<br>Nobumoto Tomioka<br>Masako Sato (P)<br>Mitsugu Yamamoto<br>Hideki Maeda<br>Sayuri Terai (Sayuri Kuwahara)<br>Hanae Tachikawa (P)<br>Katsushige Yamashiro (P)<br>Hiroaki Suzuki (P)<br>Yachiyo Iwamura (P)<br>Hanae Tachikawa (P)	National Hospital Organization<br>Hokkaido Cancer Center<br>2-3-54, Kikusui4jo, Shiraishi-ku,<br>Sapporo, Hokkaido, 003-0804,<br>Japan
Hirofumi Mukai	Toru Mukohara<br>Ako Hosono<br>Takahiro Kogawa (P)<br>Nobuaki Matsubara<br>Yoichi Naito<br>Kenichi Harano<br>Yumi Fujimoto (P)<br>Takeshi Kuwata<br>Satoshi Fujii (P)<br>Shota Kusuhara<br>Yoko Fukasawa (P)<br>Masako Inoue (P)<br>Chihiro kondoh<br>Chikako Funasaka<br>Takehiro Nakao<br>Hiromichi Nakajima<br>Misao Fukuda<br>Nobuyuki Takahashi	National Cancer Center Hospital<br>East<br>6-5-1 Kashiwanoha, Kashiwa,<br>Chiba, 277-8577, Japan
Kenjiro Aogi	Shozo Ohsumi<br>Seiki Takashima (P)<br>Mina Takahashi<br>Fumitaka Hara (P)<br>Yuichiro Miyoshi<br>Mariko Kochi<br>Michiko Yamashita	National Hospital Organization<br>Shikoku Cancer Center<br>160 Minami Umemoto-machi Ko,<br>Matsuyama, Ehime, 791-0280,<br>Japan
Naohito Yamamoto	Rikiya Nakamura<br>Toshiko Miyaki<br>Rei Matsumoto (P)<br>Arihito Yoshizumi (P)<br>Hiroto Yamamoto (P)<br>Shoko Hayama<br>Itaru Sonoda (P)	Chiba Cancer Center<br>666-2 Nitona-cho, Chuo-ku,<br>Chiba, Chiba, 260-8717, Japan

Principal Investigator<br>(P)：Previous Principal Investigator	Sub-Investigator<br>(P)：Previous Sub-Investigator	Study Conducted at Address(es)
	Tamanuki Tamaki<br>Yoshiaki Hyakutake (P)<br>Koya Fujii (P)	
Toshinari Yamashita	Takashi Yamanaka<br>Tatsuya Yoshida (P)<br>Nobuyasu Suganuma (P)<br>Haruhiko Yamazaki (P)<br>Daiji Nemoto (P)<br>Yuka Matsubara (P)<br>Tomoyuki Yokose<br>Yuko Sugawara (P)<br>Kaori Kohagura (P)<br>Soji Toda<br>Saki Okamoto<br>Ai Matsui (P)<br>Mio Yasukawa<br>Daisuke Murayama (P)<br>Saori Fujiwara<br>Akari Takahashi<br>Maya Isoda	Kanagawa Cancer Center<br>2-3-2 Nakao, Asahi-ku,<br>Yokohama, Kanagawa 241-8515,<br>Japan
Yuko Tanabe<br>Toshimi Takano (P)	Yuko Tanabe (P)<br>Chihiro Kondo (P)<br>Yukinori Ozaki (P)<br>Hidetaka Kawabata<br>Nobuko Tamura<br>Takuya Ogura (P)<br>Yoko Kobayashi<br>Jun Masuda (P)<br>Kiyo Tanaka<br>Yuji Miura<br>Michiko Kurikawa (P)<br>Toshimi Takano<br>Rika Kizawa<br>Koichi Suyama<br>Takeshi Yamaguchi	Toranomon Hospital<br>2-2-2 Toranomon, Minato-ku,<br>Tokyo, 105-8470, Japan
Koji Matsumoto	Takuma Onoe<br>Meiko Nishimura (P)<br>Shintaro Takao (P)<br>Kouichi Hirokaga<br>Kaori Tane<br>Kazuki Hashimoto (P)<br>Toshiko Sakuma<br>Hideki Sakai	Hyogo Cancer Center<br>13-70 Kitaoji-cho, Akashi Hyogo,<br>673-8558, Japan
Tsutomu Iwasa<br>Junji Tsurutani (P)	Kazuhiko Nakagawa<br>Masayuki Takeda (P)<br>Hidetoshi Hayashi<br>Kaoru Tanaka<br>Tsutomu Iwasa (P)<br>Takeshi Yoshida<br>Tatsuya Okuno (P)<br>Hiroto Ueda (P)<br>Naoki Takegawa (P)	Kindai University Hospital<br>377-2 Ono-Higashi, Osaka<br>Sayama, Osaka 589-8511, Japan

Principal Investigator<br>(P)：Previous Principal Investigator	Sub-Investigator<br>(P)：Previous Sub-Investigator	Study Conducted at Address(es)
	Yoshikane Nonagase (P)<br>Satomi Watanabe (P)<br>Hisato Kawakami<br>Koji Haratani (P)<br>Ryoji Kato (P)<br>Hitomi Sakai (P)<br>Yoshifumi Komoike<br>Kimio Yonesaka<br>Shinichiro Suzuki<br>Junji Tsurutani (P)<br>Asuka Tsuya (P)<br>Soichi Fumita (P)<br>Hiroaki Kanemura<br>Seiichiro Mitani<br>Kosuke Isomoto<br>Chihiro Sato (P)<br>Takashi Kurosaki<br>Esuteru Hirokawa (P)<br>Junko Tanizaki<br>Takayuki Takahama<br>Chiaki Inagaki<br>Yusuke Kawanaka<br>Masaki Okura (P)<br>Tomohiro Nakayama<br>Toshiaki Takakura<br>Shuichi Murata	
Eriko Tokunaga	Mayumi Ishida (P)<br>Yoshiaki Nakamura<br>Chinami Koga<br>Hideki Ichiji<br>Takanobu Masuda (P)<br>Wakako Tajiri<br>Masahiro Okamoto (P)<br>Hiroki Ueo (P)<br>Yumiko Koi	National Hospital Organization<br>Kyusyu Cancer Center<br>3-1-1 Notame, Minami-ku,<br>Fukuoka, 811-1395, Japan

Republic of Korea

Principal Investigator<br>(P)：Previous Principal Investigator	Sub-Investigator<br>(P)：Previous Sub-Investigator	Study Conducted at Address(es)
Joo Hyuk Sohn	Gun Min Kim (P)<br>Jee Hung Kim (P)<br>MinHwan Kim<br>Seul-Gi Kim (P)<br>Gun Min Kim<br>Chang Gon Kim	Severance Hospital<br>50-1 Yonsei-ro, Seodaemun-gu, Seoul, 03722, Korea
Kyung-Hun Lee<br>Seock-Ah Im (P)	Tae-You Kim (P)<br>Seock-Ah Im (P)<br>Do-Youn Oh (P)<br>Tae-Yong Kim (P)	Seoul National University Hospital<br>101 Daehak-ro, Jongno-gu, Seoul, 03080, Korea

Principal Investigator<br>(P) : Previous Principal Investigator	Sub-Investigator<br>(P) : Previous Sub-Investigator	Study Conducted at Address(es)
	Dae-Won Lee (P)<br>Shin Hye Yoo (P)<br>Yeonjoo Choi (P)<br>Ja Yoon Heo (P)<br>Mi So Kim (P)<br>Sang-A Kim (P)<br>Sock-Won Yoon (P)<br>Go-un Woo (P)<br>Joohwan Park (P)<br>Sheehyun Kim (P)<br>Jung Sun Kim (P)<br>Ji Eun Park (P)<br>Chang-Bong Yang (P)<br>Jeesun Yoon (P)<br>Seul-Bi Lee (P)<br>Eun Hee Jung (P)<br>Song Yi Park (P)<br>Minsu Kang (P)<br>Jinyong Kim (P)<br>Jieun Yang (P)<br>Jeonghwan Youk (P)	
KyungHae Jung	Jae Ho Jeong<br>Sung-Bae Kim<br>Jin-Hee Ahn<br>Jeong Eun Kim (P)<br>HyeHyun Jung	Asan Medical Center<br>88 Olympic-ro 43-gil, Songpa-gu, Seoul, 05505, Korea
Keun Seok Lee	In Hae Park (P)<br>Sung Hoon Sim (P)<br>Young Mee Kwon (P)<br>HeeJung Chae	National Cancer Center<br>323 Ilsan-ro (809 Madu 1-dong), Ilsandong-gu, Goyang-si, Gyeonggi-do, 10408, Korea
Jee Hyun Kim (P)	Se Hyun Kim (P)<br>Koung Jin Suh (P)	Seoul National University Bundang Hospital<br>82, 173 beon-gil, Gumi-ro, Bundang-gu, Seongnam-si, Gyeonggi-do, 13620, Korea
Seok Yun Kang	Mi Sun Ahn	Ajou University Hospital<br>164, World cup-ro, Yeongtong-gu, Suwon-si, Gyeonggi-do, 16499, Korea

Taiwan

Principal Investigator (P)：Previous Principal Investigator	Sub-Investigator (P)：Previous Sub-Investigator	Study Conducted at Address(es)
Yen-Shen Lu	Dwan-Ying Chang<br>Ching-Hung Lin<br>Wei-Wu Chen<br>Ming-Yang Wang<br>I-Chun Chen	National Taiwan University Hospital<br>No. 7, Chung-Shan S. Rd., Taipei City, 100225, Taiwan
Mei-Ching Liu (P)<br>Chi-Feng Chung	Nei-Min Chu<br>Chi-Feng Chung (P)	Koo Foundation Sun Yat-Sen Cancer Center<br>125, Lih-Der Road, Pei-Tou District, Taipei, 112019, Taiwan
Ling-Ming Tseng	Kuang-Liang King (P)<br>Jen-Hwey Chiu<br>Yi-Fang Tsai<br>Yen-Shu Lin<br>Ta-Chung Chao<br>Chun-Yu Liu<br>Yi-Chen Lai	Taipei Veterans General Hospital<br>201, Shih-Pai Road, Sec.2, Taipei, 112201, Taiwan

Singapore

Principal Investigator (P)：Previous Principal Investigator	Sub-Investigator (P)：Previous Sub-Investigator	Study Conducted at Address(es)
Yap Yoon Sim	Rebecca Dent<br>Beh Sok Yuen<br>Chay Wen Yee<br>Lim Hsuen, Elaine<br>Loh Wei-Jen, Kiley (P)<br>Mabel Wong<br>Chan Junjie, Jack<br>Chew Lee Lian<br>Ho Teng Swan, Juliana<br>Ng Chee Hu, Raymond<br>Huren Sivaraj (P)	National Cancer Centre Singapore<br>11 Hospital Drive, Singapore, 169610
Soo Chin Lee	Yvonne Ang<br>Joan Choo	National University Hospital

Principal Investigator<br>(P) : Previous Principal Investigator	Sub-Investigator<br>(P) : Previous Sub-Investigator	Study Conducted at Address(es)
	Matilda Lee<br>Gloria Chan<br>Natalie Ngoi<br>Anea Wong<br>Samuel Ow<br>Lim Yi Wan<br>Lim Siew Eng<br>Joline Lim	1E Kent Ridge Road, NUHS Tower Block Level 7, Singapore, 119228

Supplementary Figure 1. Trial design.

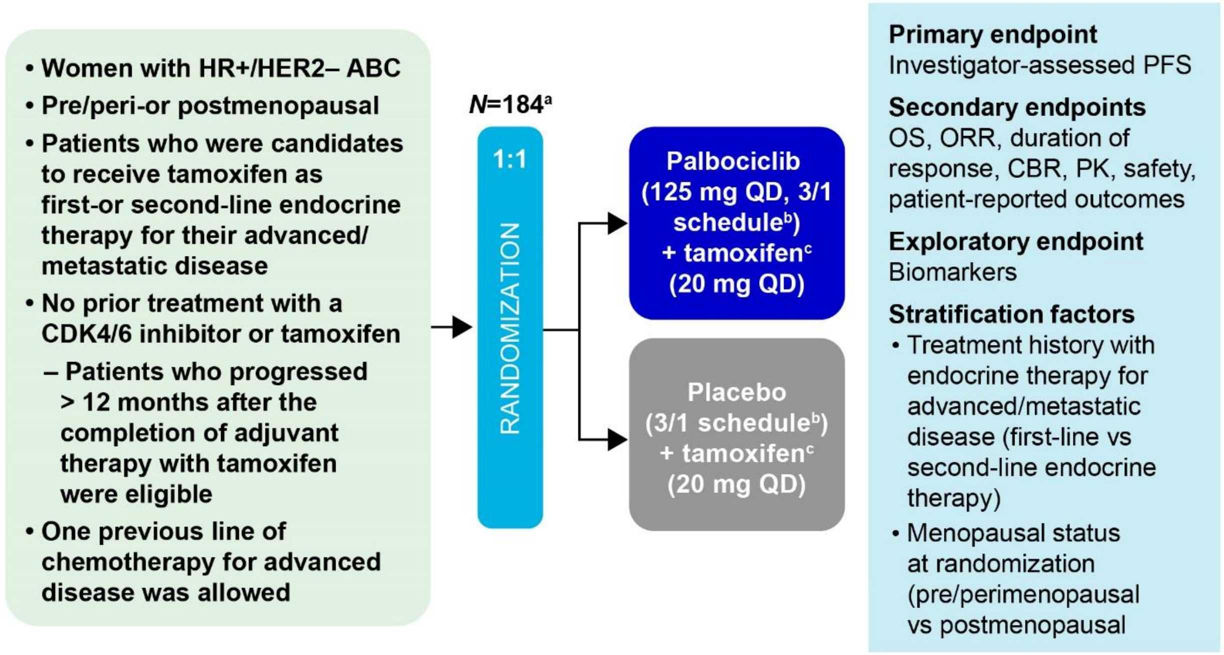

[a]Actual.
[b]3/1 schedule defined as 3 weeks on and 1 week off therapy.
[c]Pre/perimenopausal women additionally received luteinizing hormone-releasing hormone agonist (goserelin).
ABC, advanced breast cancer; CBR, clinical benefit rate; CDK4/6, cyclin-dependent kinase 4/6; HER2, human epidermal growth factor receptor 2; HR, hormone receptor; ORR, objective response rate; OS, overall survival; PFS, progression-free survival; PK, pharmacokinetics; QD, once daily.

Supplementary Table 1. Best overall response based on investigator assessment.

Treatment Group	*n*	Best Overall Response Based on Investigator Assessment, *n* (%)					Response Rate, *n* (%) (95%CI)[a]	Odds Ratio (95% CI)[b]
		CR	PR	SD	PD	NE		
Palbociclib-Tamoxifen	91	2 (2.2)	38 (41.8)	41 (45.1)	4 (4.4)	6 (6.6)	40 (44.0) (33.56–54.75)	2.02 (1.095–3.727)
Placebo-Tamoxifen	93	2 (2.2)	24 (25.8)	45 (48.4)	21 (22.6)	1 (1.1)	26 (28.0) (19.14–38.22)	

[a]Clopper Pearson method.

[b]Stratified odds ratio adjusted by the treatment history with endocrine therapy for advanced/metastatic disease and menopausal status at randomization.

CI, confidence interval; CR, complete response; NE, not evaluable; PD, progressive disease; PR, partial response; SD, stable disease.

Supplementary Table 2. Serious adiverse events related to study treatment, by severity grade and type.

Serious adverse event	Palbociclib–Tamoxifen Group (*n*=91)			Placebo–Tamoxifen Group (*n*=93)		
	Any grade, *n* (%)	Grade 3, *n* (%)	Grade 4, *n* (%)	Any grade, *n* (%)	Grade 3, *n* (%)	Grade 4, *n* (%)
**Any serious adverse events**	2 (2.2)	1 (1.1)	0	3 (3.2)	1 (1.1)	1 (1.1)
**Gastric ulcer**	1 (1.1)	0	0	0	0	0
**Peripheral swelling**	0	0	0	1 (1.1)	0	0
**Cellulitis**	1 (1.1)	1 (1.1)	0	0	0	0
**Erysipelas**	1 (1.1)	1 (1.1)	0	0	0	0
**Hypokalemia**	0	0	0	1 (1.1)	1 (1.1)	0
**Hyponatremia**	0	0	0	1 (1.1)	0	1 (1.1)
**Pulmonary embolism**	0	0	0	1 (1.1)	1 (1.1)	0

Adverse events were graded by CTCAE v4.0.
Patient with more than one adverse event within the same level of MedDRA term was counted as one in its maximum grade.
CTCAE, Common Terminology Criteria for Adverse Events; MedDRA, Medical Dictionary for Regulatory Activities.

Supplementary Table 3. Adverse events leading to treatment discontinuation, by severity grade and type.

Adverse event leading to discontinuation	Palbociclib–Tamoxifen Group (*n*=91)			Placebo–Tamoxifen Group (*n*=93)		
	Any grade, *n* (%)	Grade 3, *n* (%)	Grade 4, *n* (%)	Any grade, *n* (%)	Grade 3, *n* (%)	Grade 4, *n* (%)
**Any adverse event leading to discontinuation**	3 (3.3)	1 (1.1)	2 (2.2)	2 (2.2)	1 (1.1)	0
**Septic shock**	1 (1.1)	0	1 (1.1)	0	0	0
**Femur fracture**	1 (1.1)	1 (1.1)	0	0	0	0
**Neutrophil count decreased**	1 (1.1)	0	1 (1.1)	0	0	0
**Thyroid cancer**	0	0	0	1 (1.1)	1 (1.1)	0
**Dizziness**	0	0	0	1 (1.1)	0	0

Adverse events were graded by CTCAE v4.0.
Patient with more than one adverse event within the same level of MedDRA term was counted as one in its maximum grade.
CTCAE, Common Terminology Criteria for Adverse Events; MedDRA, Medical Dictionary for Regulatory Activities.

Supplementary Table 4. Follow-up anticancer therapy received by 3% or more of patients in either treatment group.

	Palbociclib–Tamoxifen *n*=91 *n* (%)	Placebo–Tamoxifen *n*=93 *n* (%)	Total *N*=184 *n* (%)
**Any therapy**	64 (70.3)	80 (86.0)	144 (78.3)
**Any first subsequent therapy**	64 (70.3)	80 (86.0)	144 (78.3)
Fulvestrant	11 (12.1)	7 (7.5)	18 (9.8)
Tamoxifen citrate	7 (7.7)	11 (11.8)	18 (9.8)
Abemaciclib, letrozole	3 (3.3)	8 (8.6)	11 (6.0)
Letrozole	5 (5.5)	5 (5.4)	10 (5.4)
Palbociclib, fulvestrant	3 (3.3)	6 (6.5)	9 (4.9)
Abemaciclib, fulvestrant	2 (2.2)	6 (6.5)	8 (4.3)
Anastrozole	5 (5.5)	2 (2.2)	7 (3.8)
Everolimus, exemestane	5 (5.5)	2 (2.2)	7 (3.8)
Tegafur/gimeracil/oteracil potassium	3 (3.3)	1 (1.1)	4 (2.2)
Palbociclib, letrozole	0	4 (4.3)	4 (2.2)
Paclitaxel	0	3 (3.2)	3 (1.6)
**Any second subsequent therapy**	38 (41.8)	53 (57.0)	91 (49.5)
Everolimus, exemestane	2 (2.2)	5 (5.4)	7 (3.8)
Fulvestrant	5 (5.5)	1 (1.1)	6 (3.3)
Capecitabine	1 (1.1)	5 (5.4)	6 (3.3)
Tegafur/gimeracil/oteracil potassium	4 (4.4)	1 (1.1)	5 (2.7)
Abemaciclib, fulvestrant	2 (2.2)	3 (3.2)	5 (2.7)
Paclitaxel	2 (2.2)	3 (3.2)	5 (2.7)
Exemestane	1 (1.1)	3 (3.2)	4 (2.2)
Palbociclib	0	4 (4.3)	4 (2.2)
**Any third or greater subsequent therapy**	31 (34.1)	42 (45.2)	73 (39.7)
Capecitabine	8 (8.8)	8 (8.6)	16 (8.7)
Eribulin mesylate	6 (6.6)	6 (6.5)	12 (6.5)
Everolimus, exemestane	1 (1.1)	10 (10.8)	11 (6.0)
Eribulin	4 (4.4)	6 (6.5)	10 (5.4)
Tegafur/gimeracil/oteracil potassium	5 (5.5)	2 (2.2)	7 (3.8)
Vinorelbine tartrate	4 (4.4)	3 (3.2)	7 (3.8)
Paclitaxel	4 (4.4)	2 (2.2)	6 (3.3)
Gemcitabine hydrochloride	2 (2.2)	4 (4.3)	6 (3.3)

Bevacizumab, paclitaxel	3 (3.3)	2 (2.2)	5 (2.7)
Abemaciclib, fulvestrant	3 (3.3)	1 (1.1)	4 (2.2)
Cyclophosphamide monohydrate, doxorubicin hydrochloride	1 (1.1)	3 (3.2)	4 (2.2)
Letrozole	1 (1.1)	3 (3.2)	4 (2.2)
Gemcitabine hydrochloride, paclitaxel	0	4 (4.3)	4 (2.2)

CONSORT 2010 checklist of information to include when reporting a randomised trial*

Section/Topic	Item No	Checklist item	Reported on page No
**Title and abstract**			
	1a	Identification as a randomised trial in the title	Abstract on pg 3
	1b	Structured summary of trial design, methods, results, and conclusions (for specific guidance see CONSORT for abstracts)	3
**Introduction**			
Background and objectives	2a	Scientific background and explanation of rationale	4
	2b	Specific objectives or hypotheses	4
**Methods**			
Trial design	3a	Description of trial design (such as parallel, factorial) including allocation ratio	12
	3b	Important changes to methods after trial commencement (such as eligibility criteria), with reasons	Protocol
Participants	4a	Eligibility criteria for participants	12, 13
	4b	Settings and locations where the data were collected	12
Interventions	5	The interventions for each group with sufficient details to allow replication, including how and when they were actually administered	13
Outcomes	6a	Completely defined pre-specified primary and secondary outcome measures, including how and when they were assessed	14
	6b	Any changes to trial outcomes after the trial commenced, with reasons	Protocol
Sample size	7a	How sample size was determined	15
	7b	When applicable, explanation of any interim analyses and stopping guidelines	NA
Randomisation:			
	8a	Method used to generate the random allocation sequence	13

Sequence generation	8b	Type of randomisation; details of any restriction (such as blocking and block size)	13
Allocation concealment mechanism	9	Mechanism used to implement the random allocation sequence (such as sequentially numbered containers), describing any steps taken to conceal the sequence until interventions were assigned	13
Implementation	10	Who generated the random allocation sequence, who enrolled participants, and who assigned participants to interventions	13
Blinding	11a	If done, who was blinded after assignment to interventions (for example, participants, care providers, those assessing outcomes) and how	12
	11b	If relevant, description of the similarity of interventions	NA
Statistical methods	12a	Statistical methods used to compare groups for primary and secondary outcomes	15
	12b	Methods for additional analyses, such as subgroup analyses and adjusted analyses	15
**Results**			
Participant flow (a diagram is strongly recommended)	13a	For each group, the numbers of participants who were randomly assigned, received intended treatment, and were analysed for the primary outcome	Figure 1
	13b	For each group, losses and exclusions after randomisation, together with reasons	Figure 1
Recruitment	14a	Dates defining the periods of recruitment and follow-up	13, 14
	14b	Why the trial ended or was stopped	NA
Baseline data	15	A table showing baseline demographic and clinical characteristics for each group	Table 1
Numbers analysed	16	For each group, number of participants (denominator) included in each analysis and whether the analysis was by original assigned groups	Figures
Outcomes and estimation	17a	For each primary and secondary outcome, results for each group, and the estimated effect size and its precision (such as 95% confidence interval)	5 to 7
	17b	For binary outcomes, presentation of both absolute and relative effect sizes is recommended	NA

Ancillary analyses	18	Results of any other analyses performed, including subgroup analyses and adjusted analyses, distinguishing pre-specified from exploratory	7, 8
Harms	19	All important harms or unintended effects in each group (for specific guidance see CONSORT for harms)	7, 8
**Discussion**			
Limitations	20	Trial limitations, addressing sources of potential bias, imprecision, and, if relevant, multiplicity of analyses	9 to 12
Generalisability	21	Generalisability (external validity, applicability) of the trial findings	9 to 12
Interpretation	22	Interpretation consistent with results, balancing benefits and harms, and considering other relevant evidence	9 to 12
**Other information**			3
Registration	23	Registration number and name of trial registry	
Protocol	24	Where the full trial protocol can be accessed, if available	Supplement
Funding	25	Sources of funding and other support (such as supply of drugs), role of funders	16

Citation: Schulz KF, Altman DG, Moher D, for the CONSORT Group. CONSORT 2010 Statement: updated guidelines for reporting parallel group randomised trials. BMC Medicine. 2010;8:18.

© 2010 Schulz et al. This is an Open Access article distributed under the terms of the Creative Commons Attribution License (http://creativecommons.org/licenses/by/2.0), which permits unrestricted use, distribution, and reproduction in any medium, provided the original work is properly cited.

*We strongly recommend reading this statement in conjunction with the CONSORT 2010 Explanation and Elaboration for important clarifications on all the items. If relevant, we also recommend reading CONSORT extensions for cluster randomised trials, non-inferiority and equivalence trials, non-pharmacological treatments, herbal interventions, and pragmatic trials. Additional extensions are forthcoming: for those and for up-to-date references relevant to this checklist, see www.consort-statement.org.
